# Organic Anion Transporting Polypeptide 2B1 (OATP2B1) Genetic Variants: *In Vitro* Functional Characterization and Association With Circulating Concentrations of Endogenous Substrates

**DOI:** 10.3389/fphar.2021.713567

**Published:** 2021-09-14

**Authors:** Samantha Medwid, Hayley R. Price, Daniel P. Taylor, Jaymie Mailloux, Ute I. Schwarz, Richard B. Kim, Rommel G. Tirona

**Affiliations:** ^1^ Department of Physiology & Pharmacology, University of Western Ontario, London, ON, Canada; ^2^ Division of Clinical Pharmacology, Department of Medicine, University of Western Ontario, London, ON, Canada; ^3^ Department of Oncology, Schulich School of Medicine, University of Western Ontario, London, ON, Canada

**Keywords:** drug transporter, genetic variant, endogenous substrates, organic anion transporting polypeptide 2B1 (OATP2B1), pharmacogenenomics and personalised medicine

## Abstract

Organic anion transporting polypeptide 2B1 (OATP2B1, gene *SLCO2B1*) is an uptake transporter that is thought to determine drug disposition and in particular, the oral absorption of medications. At present, the clinical relevance of *SLCO2B1* genetic variation on pharmacokinetics is poorly understood. We sought to determine the functional activity of 5 of the most common missense OATP2B1 variants (c.76_84del, c.601G>A, c.917G>A, c.935G>A, and c.1457C>T) and a predicted dysfunctional variant (c.332G>A) *in vitro*. Furthermore, we measured the basal plasma concentrations of endogenous OATP2B1 substrates, namely estrone sulfate, dehydroepiandrosterone sulfate (DHEAS), pregnenolone sulfate, coproporphyrin I (CPI), and CPIII, and assessed their relationships with *SLCO2B1* genotypes in 93 healthy participants. Compared to reference OATP2B1, the transport activities of the c.332G>A, c.601G>A and c.1457C>T variants were reduced among the substrates examined (estrone sulfate, DHEAS, CPI, CPIII and rosuvastatin), although there were substrate-dependent effects. Lower transport function of OATP2B1 variants could be explained by diminished cell surface expression. Other OATP2B1 variants (c.76-84del, c.917G>A and c.935G>A) had similar activity to the reference transporter. In the clinical cohort, the *SLCO2B1* c.935G>A allele was associated with both higher plasma CPI (42%) and CPIII (31%) concentrations, while *SLCO2B1* c.917G>A was linked to lower plasma CPIII by 28% after accounting for the effects of age, sex, and *SLCO1B1* genotypes. No association was observed between *SLCO2B1* variant alleles and estrone sulfate or DHEAS plasma concentrations, however 45% higher plasma pregnenolone sulfate level was associated with *SLCO2B1* c.1457C>T. Taken together, we found that the impacts of OATP2B1 variants on transport activities *in vitro* were not fully aligned with their associations to plasma concentrations of endogenous substrates *in vivo*. Additional studies are required to determine whether circulating endogenous substrates reflect OATP2B1 activity.

## Introduction

Organic anion transporting peptide 2B1 (OATP2B1, previously known as OATP-B, gene name *SLCO2B1*) is a member of the solute transporting carrier (SLC) superfamily. OATP2B1 is involved in the cellular uptake of a wide variety of drugs including 3-hydroxy-3-methyl-glutaryl-coenzyme A (HMG-Co-A) reductase inhibitors and fexofenadine (Kobayashi et al., 2003; Nozawa et al., 2004), as well as endogenous compounds such as steroid hormone conjugates (estrone sulfate, dehydroepiandrosterone sulfate (DHEAS), and pregnenolone sulfate), coproporphyrins (CP) and thyroid hormones (Tamai et al., 2000; Kullak-Ublick et al., 2001; Pizzagalli et al., 2003; Grube et al., 2006a; Bednarczyk and Boiselle, 2016; Shen et al., 2016; Meyer Zu Schwabedissen et al., 2018). OATP2B1 is ubiquitously expressed throughout the body in organs including intestine, liver, kidney, brain, heart, skeletal muscle, lung, placenta, pancreas and macrophages (Tamai et al., 2000; Kullak-Ublick et al., 2001; St-Pierre et al., 2002; Grube et al., 2006b; Niessen et al., 2009; Seki et al., 2009; Knauer et al., 2010; Hussner et al., 2015; Kim M. et al., 2017; Nakano et al., 2019). It is generally appreciated that intestinal OATP2B1 is involved in the oral absorption of medications as its inhibition by fruit juices is thought to reduce the bioavailability of substrate drugs including fexofenadine and celiprolol in humans (Dresser et al., 2002; Lilja et al., 2003). Indeed, pharmacokinetic studies in OATP2B1 knockout mice convincingly revealed a role of this transporter in the oral absorption of some substrate drugs, as well as a target of food- and drug-drug interactions (Medwid et al., 2019; Chen et al., 2020). Although there is significant experimental support for the relevance of intestinal OATP2B1 to drug absorption (McFeely et al., 2019), the impact of this transporter on drug distribution and elimination in other tissues where it is also expressed, remains significantly less understood (Kinzi et al., 2021).

Genetic variations and in particular, nonsynonymous single nucleotide variants (SNV) in drug transporters can be responsible for interindividual differences in drug response (Yee et al., 2018). Indeed, a SNV in the liver-specific OATP1B1 transporter (*SLCO1B1* c.521T>C), has become an established clinical pharmacogenetic marker that predicts systemic drug exposure (Niemi et al., 2011) and in some instances, treatment outcomes (SEARCH Collaborative Group et al., 2008; Trevino et al., 2009). For the most part, *in vitro* studies have consistently shown that the OATP1B1 c.521T>C (*5) variant has reduced activity (Tirona et al., 2001), which is mechanistically in keeping with the well-recognized influence on clinical pharmacokinetics and drug responses. In contrast, the pharmacological and therapeutic relevance of *SLCO2B1* genetic variation is less clear despite numerous clinical and *in vitro* studies examining the potential impacts.

Associations between the pharmacokinetics or responses of OATP2B1 substrate drugs for the most common *SLCO2B1* missense SNVs, c.935G>A and c.1457C>T (global mean allelic frequencies of 17.6 and 8.6%, respectively), have been reported in many studies, however their results have not always been consistent. For instance, with the most common *SLCO2B1* c.935G>A variant (*3 allele), montelukast plasma concentrations were lower in participants carrying the variant allele in some studies (Mougey et al., 2009; Mougey et al., 2011) but not others (Kim et al., 2013; Tapaninen et al., 2013). The *SLCO2B1* c.935G>A variant did not associate with plasma rosuvastatin concentrations in some studies (DeGorter et al., 2013; Kim TE. et al., 2017), although this genetic marker was linked to reduced lipid lowering effects. (Kim TE. et al., 2017). In prostate cancer patients undergoing androgen deprivation therapy, *SLCO2B1* c.935G>A variant carriers were compellingly shown to have shorter time to progression in different cohorts (Yang et al., 2011; Fujimoto et al., 2013; Wang et al., 2016; Hahn et al., 2019).

With respect to the *SLCO2B1* c.1457C>T variant allele and pharmacokinetic associations, contradicting studies have also been reported. For example, the *SLCO2B1* c.1457C>T variant was associated with having higher, lower or no impact on systemic exposures of fexofenadine (Akamine et al., 2010; Imanaga et al., 2011; Kashihara et al., 2017). Moreover, in one study the *SLCO2B1* c.1457C>T variant was linked to lower circulating concentrations of celiprolol (Ieiri et al., 2012) but no association was observed in another report (Kashihara et al., 2017). In a recent study, 22% lower concentration of the 3S-5R-fluvastatin enantiomer was observed in subjects with the *SLCO2B1* c.1457C>T variant, per allele (Hirvensalo et al., 2019).

*In vitro* studies have similarly provided heterogeneous results for the transport activity of OATP2B1 genetic variants. The OATP2B1 c.935G>A variant has mostly been associated with reduced transport activity, but its functional impact appears to be highly substrate- and experimental model-dependent (Nozawa et al., 2002; Ho et al., 2006; Yang et al., 2011; Nies et al., 2013; Yang et al., 2020). With the OATP2B1 c.1457C>T variant, *in vitro* studies are also conflicting with some reporting reduced transport activity (Nozawa et al., 2002; Nies et al., 2013), while for others, there was enhanced function (Ho et al., 2006; Yang et al., 2020), again with substrate-dependent effects. Taken together, because of all the divergent and inconsistent findings from clinical and biochemical studies, the potential impacts of *SLCO2B1* genetic variation to transporter activity remains to be understood.

The circulating concentrations of certain endogenous drug transporter substrates have become clinical biomarkers of transporter activity, especially in the context of predicting altered pharmacokinetics with drug-drug interactions and disease states (Rodrigues and Rowland, 2019). Indeed, coproporphyrin I (CPI) is a validated endogenous biomarker of OATP1B (OATP1B1 and OATP1B3) activity (Lai et al., 2016; Shen et al., 2016). Interestingly however, is that individuals homozygous for the reduced function *SLCO1B1* c.521T>C variant have about 2-fold higher baseline plasma CPI concentrations (Yee et al., 2018; Mori et al., 2019; Suzuki et al., 2021). Furthermore, there is 1.4-fold higher plasma estrone sulfate concentration in carriers of the *SLCO1B1* c.521T>C variant allele (van der Deure et al., 2008). These findings with *SLCO1B1* raise the possibility that the *in vivo* relevance of *SLCO2B1* genetic variation can be addressed by examining the concentrations of its circulating endogenous substrates.

In this report, we evaluated the *in vitro* transport activity of the most common OATP2B1 genetic variants in global populations [c.935G>A (*3), c.1457C>T, c.76_84del, c.917G>A, and c.601G>A] with the motivation of clarifying whether these cause functional effects. Furthermore, we explored the possibility that genetic variations in *SLCO2B1* are associated with the plasma concentrations of its endogenous substrates, namely, estrone sulfate, DHEAS, pregnenolone sulfate, CPI and CPIII. Our key findings are that circulating CPI and CPIII concentrations are greater in healthy individuals carrying the common *SLCO2B1* c.935G>A variant allele. However, there was a lack of significant impact of the OATP2B1 c.935G>A variant on substrate transport activity when tested *in vitro*.

## Materials and Methods

### Reagents

Rosuvastatin, d5-estrone sulfate, d5-DHEAS, ^15^N_4_-CPI, d8-CPIII and d6-rosuvastatin were purchased from Toronto Research Chemicals (Toronto, ON, Canada). CPI and CPIII were obtained from Frontier Specialty Chemicals (Logan, UT, United States). All other chemicals, unless otherwise stated, were obtained from Sigma-Aldrich (St. Louis, MO).

### Variant OATP2B1 Plasmid Construction

hOATP2B1 reference sequence in pcDNA3.1 (Invitrogen, Carlsbad, CA, United States) expression plasmid was prepared using methods outlined previously (Tirona et al., 2003). OATP2B1 variant expression plasmids were created using QuikChange II site-directed mutagenesis kit (Agilent Technologies; Santa Clara, CA, United States) and primers found in Supplementary Table S1, following manufacturer’s protocol. Reference OATP2B1 sequence and presence of polymorphisms were verified by Sanger sequencing.

### Transient Overexpression of OATP2B1 and Variants in Cultured Cells

Human embryonic kidney type T (HEK293T) cells were purchased from American Type Culture Collection (Manassas, VA, United States) for use in transient transfection studies. HEK293 cells are routinely used in drug transporter studies as they are efficiently transfected and express relatively low basal expression of drug transporters (Ahlin et al., 2009). Previous studies which examined the transport function of OATP2B1 genetic variants have used the HEK293 cell line for transporter overexpression (Nozawa et al., 2002; Nies et al., 2013; Yang et al., 2020). HEK293T cells were cultured in Dulbecco’s Modified Eagle Medium (DMEM) (Thermo Scientific, Grand Island, NY, United States) supplemented with 10% fetal bovine serum (FBS), 100 U/ml penicillin, and 2 mM L-glutamine (Invitrogen), at 37°C, 5% CO_2_. For HEK293T cell transport experiments, cells were grown on poly-L-lysine-coated 24-well plates. After 24 h, cells were transfected with blank insert expression plasmids (vector control) or expression plasmids containing transporter cDNA inserts (1 µg DNA/well) using Lipofectamine 3000 (Invitrogen), according to our previously described method (Medwid et al., 2019). Cells were incubated with transfection plasmids for 16 h prior to experiments.

### Solute Transport by Reference OATP2B1 and Variants in Vitro

HEK293T cells were plated onto 24-well culture plates for solute uptake experiments. Estrone sulfate, DHEAS, CPI, CPIII or rosuvastatin (each at 1 μg/ml final concentration) was dissolved in modified Krebs-Henseleit buffer (KHB) (1.2 mM MgSO_4_, 0.96 mM KH_2_PO_4_, 4.83 mM KCl, 118 mM NaCl, 1.53 mM CaCl_2_, 23.8 mM NaHCO_3_, 12.5 mM 4-[2-hydroxyethyl]-1-piperazineethanesulfonic acid, 5 mM glucose) at pH 6. Cultured cells were treated with substrates (200 µl) for 10–30 min at 37°C, 5% CO_2_. Thereafter, cells were washed three times rapidly with ice-cold phosphate-buffered saline (PBS). Cells were lysed using 200 µl of acetonitrile (for estrone sulfate, DHEAS, or rosuvastatin analyses) or 12 M formic acid (for CPI and CPIII analyses) spiked with internal standards (d5-estrone sulfate 100 ng/ml, d5-DHEAS 100 ng/ml, ^15^N_4_-CPI 100 nM, or d6-rosuvastatin 20 ng/ml). Cell lysates were centrifuged for 10 min at 13,500 rpm in a microcentrifuge and supernatants were dried in a SpeedVac (Thermo Fisher) at 45°C and resuspended in 100–200 µl mobile phase. Residues were analyzed for estrone sulfate, DHEAS, CPI, CPIII and rosuvastatin by liquid chromatography-tandem mass spectrometry (LC-MS/MS) methods described below. The specific functional activity of transfected wildtype OATP2B1 and its variants were determined after subtraction of the cellular substrate uptake of blank vector control transfected cells.

### Cell Surface Protein Biotinylation

After transfection of HEK293T cells, sulfo-NHS-SS-Biotin (Thermo Scientific) diluted 0.5 mg/ml/well in PBS containing 100 μM CaCl_2_ and 2.12 mM MgCl_2_ (PBS/Mg/Ca) was added to cells and incubated for 1 h at 4°C. Cells were then washed with ice-cold PBS/Mg/Ca containing 50 μM glycine (PBS/Mg/Ca/glycine) 3 times followed by a 20-min incubation with PBS/Mg/Ca/glycine. Cells were lysed in RIPA buffer [10 mM Tris, 150 mM NaCl, 1.27 mM EDTA, 0.1% (w/v) SDS, and 10% (v/v) Triton X-100] containing protease inhibitors and cell lysate was sonicated. Streptavidin-agarose (Thermo Scientific) was added to a proportion of cells and rocked for 1 h at room temperature. The remaining cell lysate in RIPA buffer was used to determine total protein concentrations. Streptavidin-agarose samples were then centrifuged for 3 min at 18,000 g and the pellet washed 3 times with ice-cold RIPA buffer. Pellets were subsequently rocked with 4X LDS sample buffer (Invitrogen) containing 5% 2-β-mercaptoethanol and protease inhibitors for 30 min. Thereafter, samples were centrifuged and supernatant was collected and stored until sodium dodecylsulfate - polyacrylamide gel electrophoresis (SDS-PAGE) and Western blotting.

### Western Blot

Cell surface biotinylated and total protein samples were analyzed by SDS-PAGE using 4–12% gradient gels (NuPage, Invitrogen). After transfer to polyvinylidene difluoride membranes, blots were probed with human OATP2B1 (Cat. No. H-189, Santa Cruz, Dallas, TX), GAPDH (Cat. No. sc-4772, Santa Cruz) or Na+/K+ ATPase (Cat. No. 3010S, Cell Signalling, Danvers, MA, United States) and visualized using horseradish-peroxidase labeled anti-mouse or anti-rabbit antibodies (Cell Signaling) and chemiluminescence reagent (Amersham ECL Select, GE Healthcare) on an ImageQuant LAS 500 (GE Healthcare, Mississauga, ON, Canada).

### Participants and Plasma Samples

Morning (∼8 am) blood samples were obtained after overnight fast from 93 healthy participants recruited from previously reported studies (Woolsey et al., 2016; McLean et al., 2018; Tirona et al., 2018). These studies were approved by the Human Research Ethics Board at University of Western Ontario (London, ON, Canada) and all participants provided informed written consent. Participant demographics can be found in Table 3.

### Liquid Chromatography-Tandem Mass Spectrometry

*Estrone Sulfate, Pregnenolone Sulfate and DHEAS Assay.* Plasma samples (100 μl) were combined with internal standard solution (300 μl) containing d5-estrone sulfate (100 ng/ml) and d5-DHEAS (100 ng/ml) in acetonitrile. Samples were vortexed and centrifuged at 13,000 g and 4°C for 15 min. The resulting supernatant was transferred to a microcentrifuge tube for drying in a SpeedVac. The residue was reconstituted in mobile phase (100 μl) containing 0.1% ammonium hydroxide in water and 0.1% ammonium hydroxide (90%/10%) for injection into the liquid chromatograph. Analytes were separated by liquid chromatography (Agilent 1200; Agilent; San Clara, CA, United States) using a Hypersil Gold column (50 × 3 mm, 5 μm, Thermo Fisher Scientific) following 60 µl sample injection. A mobile phase of 0.1% v/v ammonium hydroxide in water (A) and 0.1% v/v ammonium hydroxide in acetonitrile (B) was used, with an elution gradient of 10% B from 0–1.0 min, 10–90% B from 1.0–4.5 min, 90% B from 4.5–5.25 min, 90–10% B from 5.25–5.8 min and 90% B from 5.8–6.0 min, for a run time of 6 min and flow rate of 0.5 ml/min. The heated electrospray ionization source of the triple quadrupole mass spectrometer (Thermo TSQ Vantage; Thermo Fisher Scientific) was operated in negative mode (4000 V, 350°C) with collision energy set at 25 V. Additional ionization source conditions used were as follows: 40 arbitrary units for sheath gas pressure, 15 arbitrary units for auxiliary gas pressure and 350°C for capillary temperature. Selected reaction monitoring for estrone sulfate, d5-estrone sulfate, DHEAS, d5-DHEAS, and pregnenolone sulfate was performed using mass transitions 349.2→268.3 m/z, 354.1→273.4 m/z, 367.1→97.0 m/z, 372.1→98.0 m/z, and 395.1→97.0 m/z, respectively. Estrone sulfate/d5-estrone sulfate, DHEAS/d5-DHEAS and pregnenolone sulfate had retention times of 2.84, 2.91, and 3.13 min, respectively. Calibration samples containing estrone sulfate 0–4 ng/ml, pregnenolone sulfate 0–4,000 ng/ml and DHEAS 0–4,000 ng/ml were prepared in PBS from ethanol stock solutions and processed similarly as above.

*CPI and CPIII Assay.* CPI concentrations were measured according to a published method (Lai et al., 2016) with modifications. Plasma samples (200 μl) were combined with internal standard solution (100 μl) containing d8-CPIII 1.5 μmol/ml in 12 M formic acid. Ethyl acetate (1 ml) was combined, and samples were vortexed for 1 min and centrifuged at 13,000 g and 4°C for 15 min. The resulting organic layer (760 μl) was transferred to a microcentrifuge tube for drying in a SpeedVac. The residue was reconstituted in mobile phase (100 μl) containing 0.1% formic acid in water and 0.1% formic acid in acetonitrile (80%/20%) for injection into the liquid chromatograph. Solutes were separated on a Zorbax Eclipse Plus C18 column (100 mm × 2.1 mm, 1.8 μm). A mobile phase of 0.1% formic acid in water (A) and 0.1% formic acid in acetonitrile (B) was used, with an elution gradient of 20% B from 0–0.5 min, 20–71% B from 0.5–9 min, 71–98% B from 9–10 min, 98% B from 10–10.25, 98–20% B from 10.25-5–11.25 min and 20% B from 11.25–12.5 min, for a run time of 12.5 min and flow rate of 0.2 ml/min. Mass spectrometry detection was carried out on a TSQ Vantage triple-quadrupole instrument set in positive mode for detection of CPI/CPIII, d8-CPIII and ^15^N_4_-CPI with transitions 655.4 → 596.4 m/z, 659.3 → 600.3 m/z and 663.0 → 602.4 m/z. CPI/^15^N_4_-CPI and CPIII/d8-CPIII eluted at 8.29 and 8.54 min, respectively. Calibration samples containing CPI 0–10 nM and CPIII 0–1 nM were prepared in PBS from DMSO stock solutions and processed similarly as above. All experiments and analytical procedures involving CPI and CPIII were performed under low light conditions.

*Rosuvastatin Assay.* Analytes were separated by liquid chromatography using Hypersil Gold (50 × 3 mm, 5 µm) following 50 µl sample injection. A mobile phase of 0.1% v/v formic acid in water (A) and 0.1% v/v formic acid in acetonitrile (B) was used, with an elution gradient of 25% B from 0–1.0 min, 25–40% B from 1.0–6.0 min, 40–25% B from 6.0–7.0 min, and 25% B from 7.0–8.0 min, for a run time of 8 min and flow rate of 0.5 ml/min. The heated electrospray ionization source of the TSQ Vantage triple-quadrupole mass spectrometer was operated in positive mode (4500 V, 350°C) with collision energy set at 25 V. Additional ionization source conditions used were as follows: 40 arbitrary units for sheath gas pressure, 15 arbitrary units for auxiliary gas pressure and 350°C for capillary temperature. Selected reaction monitoring for rosuvastatin and d6-rosuvastatin was performed using mass transitions 482.1→258.2 m*/z,* and 488.0→264.3 m*/z*, respectively. Rosuvastatin and d6-rosuvatatin had a retention time of 4.6 min.

#### Genotyping

Volunteers were genotyped by TaqMan allelic discrimination assay (Applied Biosystems, Foster City, CA, United States) for *SLCO2B1* c.76_84del (rs72408262; C_99453792_10), *SLCO2B1* c.601G>A (rs35199625; C_25606765_20), *SLCO2B1* c.917G>A (rs78825186; C_105413676_20), *SLCO2B1* c.935G>A rs12422149; C_3101331_10), *SLCO2B1* c.1457C>T (rs2306168; C_16193013_20), *SLCO1B1* c.388A>G (rs2306283; C_1901697_20), *SLCO1B1* c.521C>T (rs4149056; C_30633906_10), *ABCG2* (Breast Cancer Resistance Protein, BCRP) c.421>A (rs2231141; C_15854163_70), *CYP* (Cytochrome P450) 2C9*2 (rs1799853; C_25625805_10), CYP2C9*3 (rs1057910; C_27104891_10), *ABCC2* (Multidrug Resistance Protein 2, MRP2) c.1249G>A (rs2273697; C_22272980_20) and *ABCC2* c.-24C>T (rs717620; C_2814642_10).

#### Statistics

Unpaired, two-tailed, student’s t-test was used to assess differences between the transport activities of variants and reference OATP2B1. Univariate analysis with unpaired student’s t-test was used to compare plasma endogenous OATP2B1 substrate concentrations among wildtype and variant carriers (heterozygous and homozygous). Multiple linear regression was used to determine the contributions of participant genotypes and demographic variables to the log-transformed plasma endogenous OATP2B1 substrate concentrations. *A priori* statistical significance was set at a *p*-value of <0.05. All statistical analysis was conducted using GraphPad Prism 9 (La Jolla, CA, United States).

## Results

### Transport Activity of OATP2B1 Genetic Variants

**FIGURE 1:**
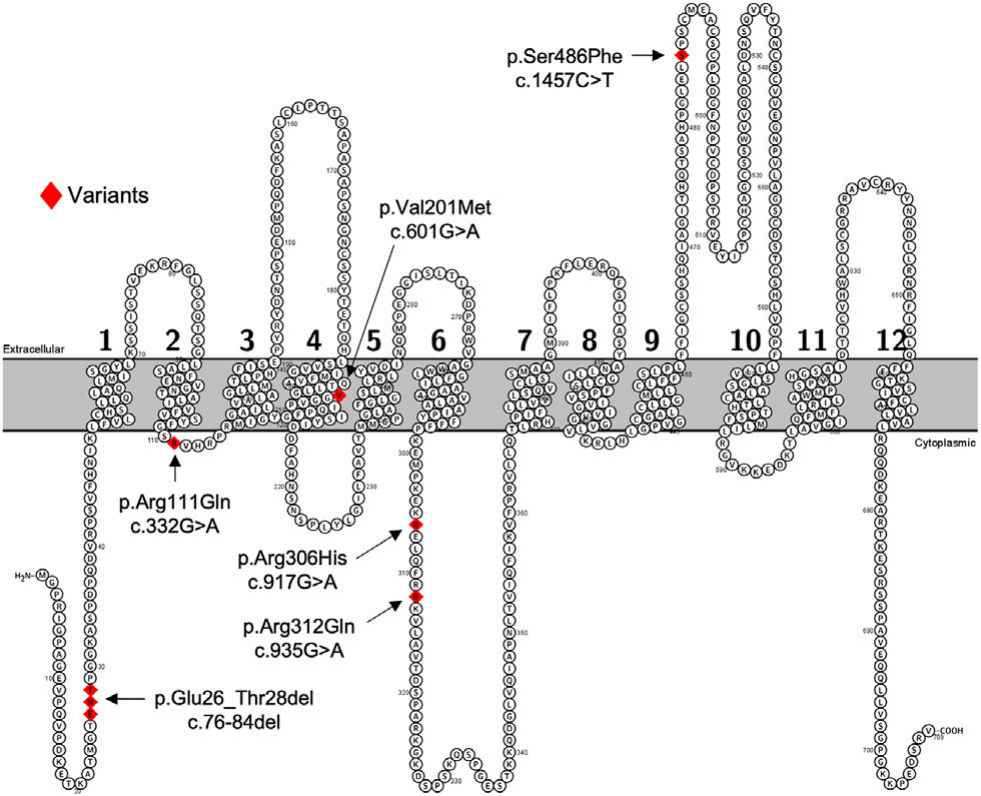
Predicated 2-D structure of OATP2B1 full length transcriptional variant. Genetic variants of interest are highlighted in red and indicated by arrows with residue number and amino acid change. The predicted 2-dimensional membrane topology model of OATP2B1 was generated using Protter interactive protein visualization software (https://wlab.ethz.ch/protter/start/).

**FIGURE 2:**
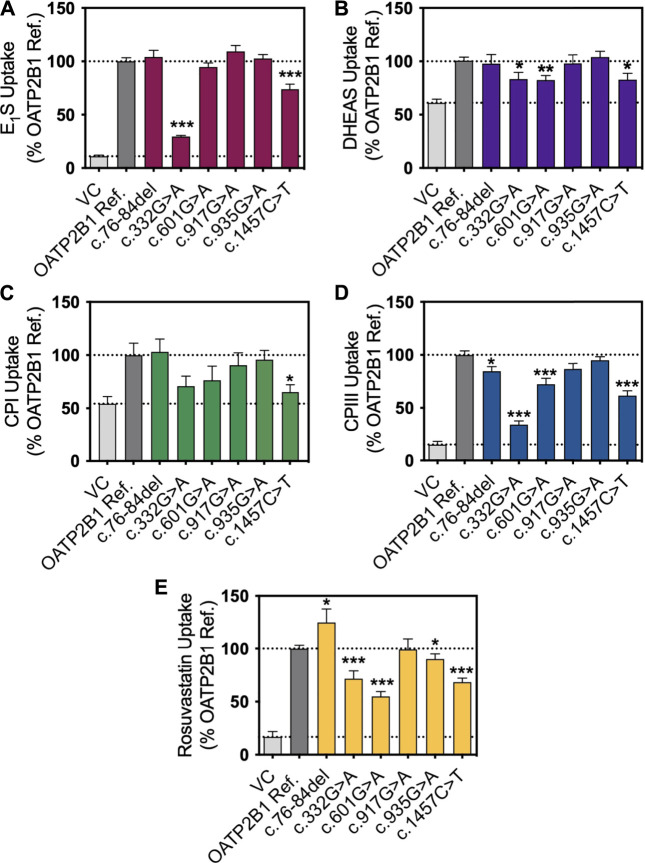
*In vitro* transport activity of OATP2B1 genetic variants with substrates. Cellular accumulation of **(A)** estrone sulfate, (E_1_S) (1 μg/ml, *n* = 3), **(B)** dehydroepiandrosterone sulfate (DHEAS) (1 μg/ml, *n* = 4), **(C)** coproporphyrin (CP) I (1 μg/ml, *n* = 3), **(D)** CPIII (1 μg/ml, *n* = 3) and **(E)** rosuvastatin (1 μg/ml, *n* = 3) in HEK293T cells were transiently transfected with vector control (VC), OATP2B1 reference and OATP2B1 variants after incubation for 10 min (E_1_S, DHEAS, CPIII and rosuvastatin) or 30 min (CPI) in Krebs-Henseleit buffer (KHB) at pH 6. Results are shown as mean ± SEM, *p < 0.05, **p < 0.01, ***p < 0.001.

We selected the five most common nonsynonymous OATP2B1 genetic variants with global allelic frequencies greater than 1% for *in vitro* functional assessment: c.76_84del, c.601G>A, c.917G>A, c.935G>A, and c.1457C>T (Table 1; Figure 1). Predicted deleteriousness or pathogenicity for the common OATP2B1 genetic variants based on computational ensemble models are shown in Table 1. The Combined Annotation Dependent Depletion (CADD) scores range in value from 0 to 100, with greater values reflecting higher probability of deleteriousness of a variant. The Rare Exom Variant Ensemble Learner (REVEL) and Meta-Logistic-Regression (MetaLR) models provide scores with values ranging from 0 to 1, with higher values predicting pathogenicity/deleteriousness. We included another rare genetic variant, OATP2B1 c.332G>A (global allelic frequency 0.0014) in the *in vitro* study as a potential positive (deleterious) control with high CADD, REVEL and MetaLR scores (Table 1). The OATP2B1 c.601G>A variant was the only other variant that the *in silico* models predict to be potentially deleterious/pathogenic. The transport activities of the OATP2B1 variants were determined by assessing cellular accumulation of the endogenous substrates estrone sulfate, DHEAS, CPI, CPIII as well as the substrate drug rosuvastatin, in transiently transfected cells. OATP2B1-mediated cellular accumulation of substrates was evidenced by 9.5-, 1.5-, 2.0-, 5.2- -and 6.5-fold greater cellular uptake for estrone sulfate, DHEAS, CPI, CPIII and rosuvastatin, respectively, when compared to blank vector control cells (Figure 2). The following summarizes the OATP2B1 variants with altered transport compared to wildtype according to substrate. OATP2B1-mediated estrone sulfate transport was significantly lower with OATP2B1 variants c.332G>A (79.2%) and c.1457C>T (29.3%) (Figure 2A). The variants c.332G>A, c.601G>A and c.1457C>T had lower OATP2B1-mediated DHEAS cellular accumulation by 43.4, 45.9 and 45.1%, respectively (Figure 2B). OATP2B1-mediated CPI uptake was lower by 75.9% with the c.1457C>T variant compared to reference (Figure 2C). For CPIII, there was lower OATP2B1-mediated transport for variants c.76-84del (18.2%), c.332G>A (77.4%), c.601G>A (32.5%), c.1457C>T (45.6%) compared to reference (Figure 2D). OATP2B1 c.76-84del had greater OATP2B1-mediated rosuvastatin cellular accumulation by 25%, while c.332G>A, c.601G>A, c.935G>A and c.1457C>T had lower transporter-mediated rosuvastatin cellular accumulation by 28.3, 45.0, 9.9, and 31.6%, respectively (Figure 2E). Across all substrates, the OATP2B1 c.1457C>T variant was found to have reduced transport activity compared to OATP2B1 reference. Lower transport activity was also generally observed for the OATP2B1 c.332G>A and c.601G>A variants, however, this was not statistically significant for all substrates. Overall, the OATP2B1 c.76-84del, c.917G>A and c.935G>A variants were not particularly different in transport activity compared to the reference transporter.

**TABLE 1:** *SLCO2B1* SNVs studied and their allele frequencies.

Designation	Nucleotide change	Protein change	CADD score^a^	REVEL score^b^	MetaLR score^b^	Minor allele frequency^c^			
						Global	Caucasian	African	East asian
rs60113013	c.76_84del	p.Glu26_Thr28del	14.9	NA	NA	0.0293	0.0187	0.0032	0.1021
rs142693902	c.332G>A	p.Arg111Gln	27.8	0.632	0.436	0.0014	0.0007	0.0001	0.0000
rs35199625	c.601G>A	p.Val201Met	22.8	0.149	0.01	0.0198	0.0124	0.0022	0.0577
rs78825186	c.917G>A	p.Arg306His	9.4	0.111	0.141	0.0146	0.0226	0.0036	0.0001
rs12422149	c.935G>A	p.Arg312Gln	7.7	0.026	0	0.1759	0.1036	0.0910	0.3261
rs2306168	c.1457C>T	p.Ser486Phe	12.0	0.022	0	0.0826	0.0244	0.3365	0.2250

a Combined Annotation Dependent Depletion (CADD) score was obtained from https://cadd.gs.washington.edu.

b Rare Exome Variant Ensemble Learner (REVEL) and MetaLR score was obtained from https://m.ensembl.org.

c Allele frequencies sourced from gnomAD database, https://gnomad.broadinstitute.org.

NA, not applicable.

### Estrone Sulfate and CPIII Transport Kinetics by OATP2B1 Genetic Variants

OATP2B1-mediated transport kinetics were further evaluated for the nonsynonymous variants with estrone sulfate and CPIII. Correcting for cellular accumulation of solutes in the vector control cells, the maximal uptake rates (V_max_), affinities (K_m_) and estimated uptake clearance (V_max_/K_m_) for OATP2B1 reference and variants are shown in Table 2. With estrone sulfate transport, the V_max_ and K_m_ values for OATP2B1 variants c.332G>A and c.1457C>T could not be determined as saturable kinetics were not evident. Assuming non-saturable, linear OATP2B1 transport, the c.332G>A and c.1457C>T variants had markedly reduced uptake clearance than reference OATP2B1. For CPIII, the OATP2B1 c.332G>A variant had clearly altered transport kinetics compared to reference OATP2B1, with a reduction of V_max_ by 73%.

**TABLE 2:** Estrone Sulfate and CPIII transport kinetics by OATP2B1 and its genetic variants.

	Variant	V_max_ ^a^ (pmol∙mg protein^−1^∙min^−1^)	K_m_ ^a^ (µM)	CL (V_max_/K_m_) (µL∙mg protein^−1^ min^−1^)
Estrone Sulfate	OATP2B1 Ref	91.6 ± 5.2	5.9 ± 1.2	15.6
	c.76-84del	70.2 ± 8.1	4.1 ± 1.8	17.0
	c.332G>A	N.D.	N.D.	0.25^b^
	c.601G>A	68.1 ± 6.8	4.0 ± 1.6	17.1
	c.917G>A	46.2 ± 3.9	1.9 ± 0.7	24.8
	c.935G>A	63.9 ± 5.1	2.8 ± 1.0	22.5
	c.1457C>T	N.D.	N.D.	0.38^b^
CPIII	OATP2B1 Ref.	25.5 ± 1.5	0.034 ± 0.011	743
	c.76-84-del	54.8 ± 5.2	0.051 ± 0.025	1,069
	c.332G>A	6.8 ± 0.8	0.055 ± 0.034	125
	c.601G>A	40.4 ± 4.9	0.052 ± 0.033	775
	c.917G>A	62.7 ± 8.0	0.058 ± 0.038	1,077
	c.935G>A	40.8 ± 3.1	0.066 ± 0.027	629
	c.1457C>T	40.5 ± 4.1	0.062 ± 0.032	649

a Mean ± standard error of estimate.

b Estimated uptake clearance based on linear regression; N.D., not determined.

### Cell Surface Expression of OATP2B1 Variants

**FIGURE 3:**
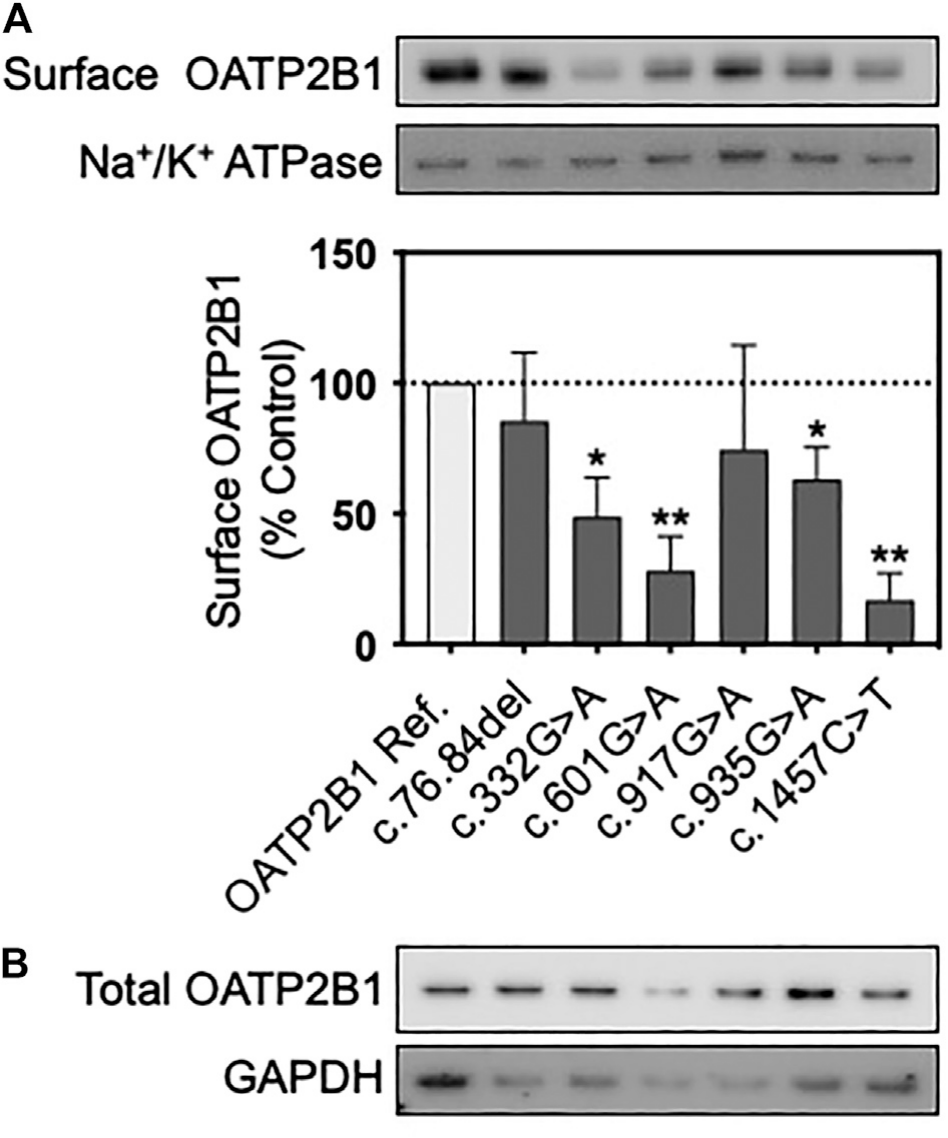
Protein expression of OATP2B1 genetic variants. Representative western blots of **(A)** cell surface and **(B)** total OATP2B1 protein expression in HEK293T cells transfected with OATP2B1 reference and OATP2B1 genetic variants. Western blot analysis of surface OATP2B1 protein expression was normalized to Na^+^/K^+^ ATPase. Results are shown as mean ± SEM (*n* = 3), *p < 0.05, **p < 0.01.

Total and cell surface protein expression of OATP2B1 reference and variants in transfected HEK293T cells were examined by western blot. Cell-surface expression of OATP2B1 was absent in blank vector transfected HEK293T cells (Supplementary Figure S1). When normalized to Na^+^/K^+^ ATPase, cell surface protein expression of OATP2B1 c.332G>A, c.601G>A, c.935G>A and c.1457C>T were decreased significantly by 51, 72, 37, and 83% compared to OATP2B1 reference, respectively (Figure 3; Supplementary Figure S1).

### Study Cohort for Circulating OATP2B1 Substrates

Plasma samples were obtained from 93 healthy volunteers for analysis. The median age was 25, 40.9% were male and the mean weight was 69.8 kg. Of the 93 participants, 69 were Caucasian, 20 East Asian, and 4 African. Allelic frequencies of each *SLCO2B1* variant in the cohort were 0.027, 0.016, 0.027, 0.123, and 0.118 for c.76-84del, c.601G>A, c.917G>A, c.935G>A and c.1457C>T, respectively (Table 3). No deviations from Hardy-Weinberg were seen for *SLCO2B1* genotypes. The allelic frequencies for *SLCO2B1* variants in the study cohort differed by race (Table 3) and were comparable to that reported in the Genome Aggregation Database (gnomAD) database (Karczewski et al., 2020) (Table 1). For example, the *SLCO2B1* c.935G>A and c.1457C>T variants were more frequent in East Asian than Caucasian participants (Table 3).

**TABLE 3:** Study participant demographics (*n* = 93).

Age, median (range)	25 (18–62)			
Sex, N (%)				
Male	38 (40.9)			
Female	55 (59.1)			
Weight (kg) (range)^a^	69.8 (43.3–108.9)			
Race, N				
African	4			
East Asian	20			
Caucasian	69			
Allelic Frequency	Entire Cohort	African	East Asian	Caucasian
*SLCO2B1* c.76-84del	0.027	0.000	0.050	0.022
*SLCO2B1* c.601G>A	0.016	0.000	0.050	0.007
*SLCO2B1* c.917G>A	0.027	0.000	0.000	0.036
*SLCO2B1* c.935G>A	0.123	0.250	0.350	0.051
*SLCO2B1* c.1457C>T	0.118	0.250	0.450	0.014
*SLCO1B1* c.388A>G	0.387	0.375	0.400	0.399
*SLCO1B1* c.521T>C	0.172	0.125	0.075	0.203

a Obtained for 79 of 93 participants.

### Effects of Demographic Factors on Plasma Endogenous OATP2B1 Substrate Concentrations

**FIGURE 4:**
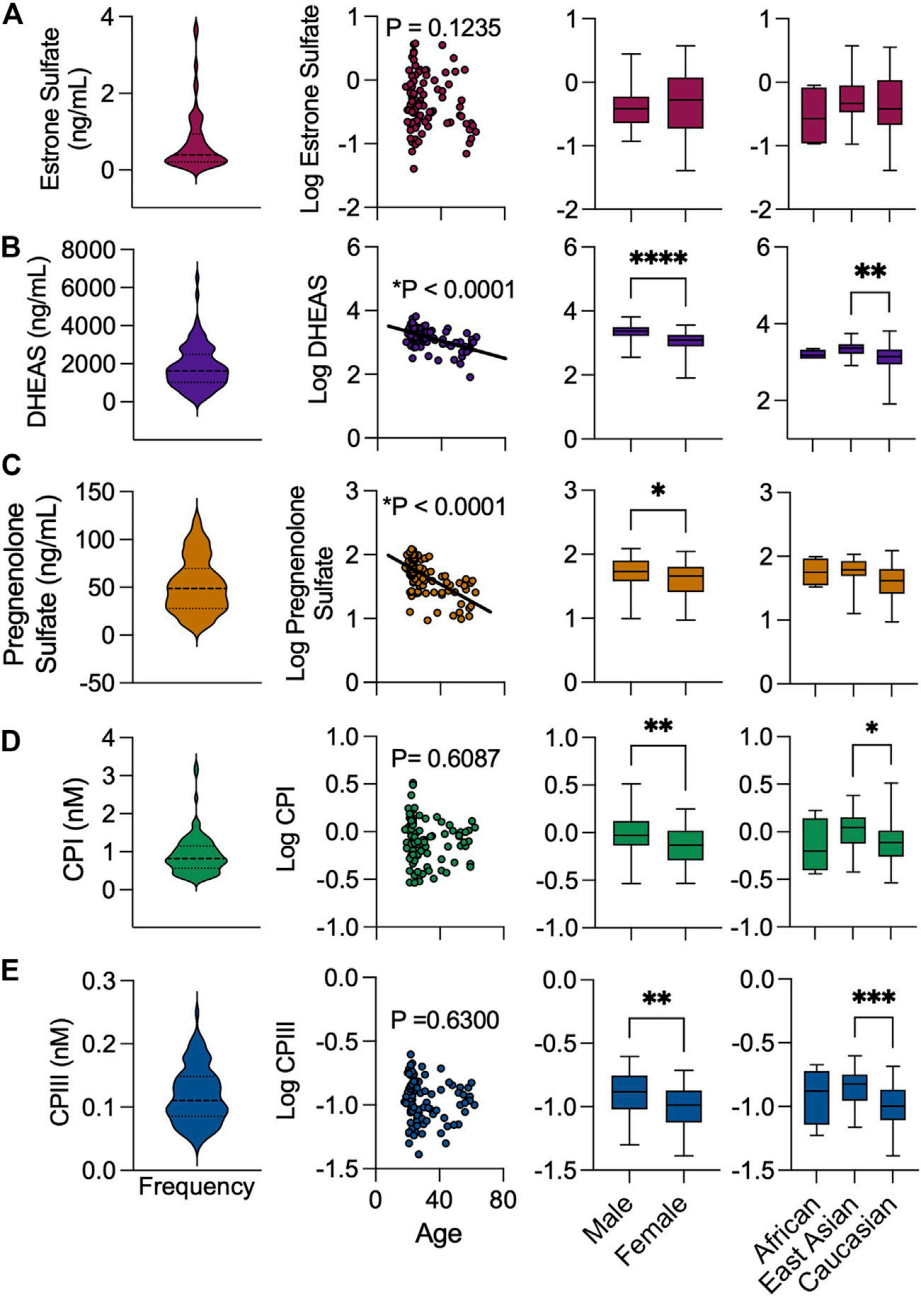
Cohort distribution of endogenous biomarkers levels by baseline demographics. Frequency distribution of **(A)** estrone sulfate, **(B)** DHEAS, **(C)** pregnenolone sulfate **(D)** CPI and **(E)** CPIII. Association of endogenous substrates with age, sex, and ethnicity. Box and whiskers plots are shown as mean (line), 25th and 75th percentile (box) and range (whiskers) *p < 0.05, **p < 0.01, ***p < 0.001, ****p < 0.0001.

Median plasma concentrations (range) of estrone sulfate, DHEAS, pregnenolone sulfate, CPI and CPIII were 0.73 ng/ml (0.04–3.74 ng/ml), 1826 ng/ml (82–6,515 ng/ml), 52.1 ng/ml (9.4–112.3 ng/ml), 0.92 nM (0.29–3.25 nM) and 0.12 nM (0.04–0.21 nM), respectively (Figure 4). Univariate analyses were performed to compare OATP2B1 endogenous substrate concentrations with demographic factors (age, sex, race). Estrone sulfate concentrations were not associated with age, sex, or race (Figure 4A). Lower DHEAS concentrations were observed with increasing age as was for female compared to male sex, and for Caucasian compared to East Asian race (Figure 4B). Similarly, younger age and male sex was associated with greater concentrations of pregnenolone sulfate (Figure 4C). Lastly, CPI and CPIII concentrations were not associated with age, however, the levels of both compounds were greater in males compared to females, and in East Asians compared to Caucasians (Figures 4D,E).

### Univariate Analysis of Genetic Variations on Plasma Endogenous OATP2B1 Substrate Concentrations

We examined whether *SLCO2B1* variants c.76-84del, c.601G>A, c.917G>A, c.935G>A, and c.1457C>T were associated with plasma concentrations of OATP2B1 endogenous substrates. The *SLCO2B1* variant c.332G>A was not genotyped in this cohort because the expected minor allelic frequency was less than 0.01% (Table 1). Pairwise comparisons showed greater plasma DHEAS (by 40%) and pregnenolone sulfate (by 57%) concentrations in participants carrying *SLCO2B1* c.1457C>Talleles (Table 4). The *SLCO2B1* c.935G>A allele was associated with higher plasma concentrations of CPI and CPIII by 43 and 46%, respectively (Table 4). Additionally, the *SLCO2B1* c.917G>A allele was associated with a 33% lower CPIII plasma levels (Table 4).

**TABLE 4:** Univariate Analyses of *SLCO2B1* gene variants with circulating endogenous substrate concentrations.

Variant	*SLCO2B1* c.76-84del		*SLCO2B1* c.601G>A		*SLCO2B1* c.917G>A		*SLCO2B1* c.935G>A		*SLCO2B1* c.1457C>T	
Carrier status^a^	NC	C	NC	C	NC	C	NC	C	NC	C
N	88	5	90	3	88	5	72	21	76	17
Estrone Sulfate (ng/ml)	0.71 (±0.08)	1.00 (±0.69)	0.71 (±0.08)	1.40 (±1.17)	0.74 (±0.09)	0.50 (±0.09)	0.75 (±0.09)	0.66 (±0.18)	0.68 (±0.08)	0.95 (±0.29)
DHEAS (ng/ml)	1836 (±123)	1,652 (±355)	1827 (±120)	1792 (±584)	1856 (±122)	1299 (±337)	1721 (±138)	2149 (±209)	1701 (±121)	2388* (±318)
Pregnenolone Sulfate (ng/ml)	52 (±3)	56 (±16)	52 (±3)	68 (±26)	53 (±3)	32 (±5)	49 (±3)	63 (±7)	47 (±3)	74** (±6)
CPI (nM)	0.92 (±0.06)	0.95 (±0.22)	0.92 (±0.05)	1.04 (±0.39)	0.93 (±0.06)	0.76 (±0.21)	0.84 (±0.06)	1.20** (±0.10)	0.92 (±0.06)	0.94 (±0.10)
CPIII (nM)	0.12 (±0.01)	0.12 (±0.02)	0.12 (±0.01)	0.15 (±0.02)	0.12 (±0.01)	0.08* (±0.01)	0.11 (±0.00)	0.16** (±0.01)	0.11 (±0.01)	0.14 (±0.01)

a NC, non-carrier; C, Carrier (heterozygotes + homozygotes).

Mean ± S.E.M.

*p < 0.05, **p < 0.01.

Since the OATP2B1 endogenous substrates (estrone sulfate, DHEAS, CPI or CPIII) measured in plasma are also substrates of other transporters (e.g., OATP1B1, MRP2 and BCRP) or subject to drug metabolism (e.g., CYP2C9), we examined their possible associations with common SNPs in these genes (Zhai et al., 2011; Dudenkov et al., 2017; Muller et al., 2018) by pairwise comparisons. *SLCO1B1* c.388A>G was associated with higher pregnenolone sulfate levels (by 47%) but not significantly for estrone sulfate, DHEAS, CPI, or CPIII concentrations (Supplementary Table S2 **)**. Likewise, *SLCO1B1* c.521T>C, *ABCG2* (BCRP) c.421C>A, CYP2C9*2, CYP2C9*3, *ABCC2* (MRP2) c.1248G>A and *ABCC2* c.-24C>T were not significantly associated with any of the endogenous substrates investigated (Supplementary Table S2).

### Multivariable Analysis of *SLCO2B1* Genetic Variations on Plasma Endogenous OATP2B1 Substrate Concentrations

Multivariable linear regression analyses were performed to determine whether *SLCO2B1* variant were associated with plasma concentrations of each of the OATP2B1 endogenous substrates. For each model, demographic variables were included such as sex, race and age, particularly when associations were found in univariate analyses. Furthermore, the clinically relevant *SLCO1B1* c.388A>G and *SLCO1B1* c.521C>T alleles were included into models because the measured solutes are also OATP1B1 substrates and for some solutes (e.g., estrone sulfate and CPI), associations with these genotypes have been previously reported. The final models with parameter estimates are shown in Table 5.

**TABLE 5:** Multivariable linear regression models for circulating endogenous substrates of OATP2B1.

Model	Variable	Coefficient	*p*-Value
Log (Estrone Sulfate) (R^2^ = 0.047)	Intercept	−0.3522	
	Sex^a^	−0.05838	0.5320
	*SLCO2B1* c.935G>A^b^	−0.02600	0.8227
	*SLCO2B1* c.1457C>T^b^	0.07600	0.5559
	*SLCO1B1* c.388A>G^b^	−0.07181	0.5051
	*SLCO1B1* c.521T>C^b^	0.2094	**0.0525**
Log (DHEAS) (R^2^ = 0.491)	Intercept	3.321	
	Sex^a^	0.2882	**<0.0001**
	Age	−0.01091	**<0.0001**
	*SLCO2B1* c.935G>A^b^	0.06765	0.2898
	*SLCO2B1* c.1457C>T^b^	0.09664	0.1906
	*SLCO1B1* c.388A>G^b^	0.05940	0.3426
	*SLCO1B1* c.521T>C^b^	−0.01988	0.7349
Log (Pregnenolone Sulfate) (R^2^ = 0.451)	Intercept	1.809	
	Sex^a^	0.1168	**0.0121**
	Age	−0.01001	**<0.0001**
	*SLCO2B1* c.935G>A^b^	0.02807	0.6161
	*SLCO2B1* c.1457C>T^b^	0.1624	**0.0135**
	*SLCO1B1* c.388A>G^b^	0.05564	0.1635
	*SLCO1B1* c.521T>C^b^	0.07223	0.3116
Log (CPI) (R^2^ = 0.271)	Intercept	−0.2455	
	Sex^a^	0.1197	**0.0063**
	Race^c^	0.1109	0.1298
	*SLCO2B1* c.935G>A ^b^	0.1512	**0.0093**
	*SLCO2B1* c.1457C>T^b^	−0.06612	0.3692
	*SLCO1B1* c.388A>G^b^	0.05354	0.2836
	*SLCO1B1* c.521T>C^b^	0.07340	0.1387
Log (CPIII) (R^2^ = 0.340)	Intercept	−1.038	
	Sex^a^	0.1084	**0.0007**
	Race^c^	0.07965	0.1410
	*SLCO2B1* c.917G>A^b^	−0.1452	**0.0370**
	*SLCO2B1* c.935G>A^b^	0.1164	**0.0057**
	*SLCO2B1* c.1457C>T^b^	−0.004622	0.9303
	*SLCO1B1* c.388A>G^b^	0.006791	0.8509
	*SLCO1B1* c.521T>C^b^	−0.01293	0.7182

a Males compared to reference (Females).

b Carriers (heterozygotes + homozygotes) compared to reference (Non-carriers).

c East Asian compared to reference (Caucasians).

In the model for estrone sulfate, there was an association of the *SLCO1B1 *c.521C>T allele with 62% higher plasma concentrations (*p* = 0.053) when the model was adjusted for sex and included other *SLCO2B1/SLCO1B1* genotypes. It is notable that variables included in the model poorly explained the interindividual variability in circulating estrone sulfate as R^2^ was 0.047.

For DHEAS, 49% of variation in circulating concentrations could be explained by a model that includes the variables of sex, age, and *SLCO2B1/SLCO1B1* genotypes. Sex and age were variables that were significantly associated with DHEAS concentrations. The model predicts males have 94% higher DHEAS concentrations, while advancing age results in decreasing plasma DHEAS, with a 22% lower level for each decade. Although *SLCO2B1* c.1457C>T was associated with DHEAS concentrations in univariate analysis, this was no longer found when adjusting for sex and age.

About 45% of the variability in circulating pregnenolone sulfate concentration was explained by a model that considers sex, age and *SLCO2B1/SLCO1B1* genotypes. Males are predicted to have 31% greater pregnenolone concentrations than females (*p* = 0.012) and increasing age significantly contributes to decreasing circulating levels (*p* < 0.0001). The *SLCO1B1* c.388A>G variant did not associate with pregnenolone sulfate concentrations as previously found in univariate analysis when adjusting for other variables. Interestingly, *SLCO2B1* c.1457C>T variant carriers continue to be associated with higher (45%, *p* = 0.014) pregnenolone sulfate concentrations with the multivariable model.

In the multivariable model for CPI, male sex is predicted to have 32% higher circulating concentrations than female sex (*p* = 0.006). Carriers of the *SLCO2B1* c.935G>A variant are predicted to have 42% greater plasma CPI levels (*p* = 0.009). There was no longer a significant association with race that was found in the univariate analysis for CPI concentrations. Furthermore, the *SLCO1B1* c.521T>C was not significantly associated with CPI levels. Altogether, approximately 27% of the variability in CPI could be explained by the model.

With the multivariable model for CPIII, female sex was significantly associated with lower CPIII concentrations by 22%. Again, race no longer was associated with circulating CPIII with multivariable regression analysis as was previously noted in the simple pairwise comparison. The *SLCO2B1* c.935G>A variant is predicted to result in 31% greater plasma CPIII (*p* = 0.006), while possession of the *SLCO2B1* c.917G>A variant was associated with 28% lower CPIII (*p* = 0.037). Approximately 35% of the variability in plasma CPIII could be explained by the model.

## Discussion

OATP2B1 is considered an emerging transporter with clinical importance according to the International Transporter Consortium (Zamek-Gliszczynskiet al., 2018) and it has been argued that this transporter is deserving of greater attention (McFeely et al., 2019; Kinzi et al., 2021). Indeed, OATP2B1 seems to be involved in the oral absorption of medications and is the target of drug interactions in the intestine (McFeely et al., 2019; Medwid et al., 2019). Nevertheless, additional evidence to support or refute roles for OATP2B1 in drug disposition and in physiological functions is needed (Bednarczyk and Sanghvi, 2020; Kinzi et al., 2021). For several drug transporters such as OATP1B1, Organic Cation Transporter 1 (OCT1) and BCRP, the occurrence of functional genetic variations that influence drug and endobiotic disposition has helped to firmly establish their clinical relevance. But for OATP2B1, there have been many inconsistencies in the effects of common missense genetic variants on the plasma concentrations of presumed substrate drugs. Furthermore, the effects of these nonsynonymous genetic variants on OATP2B1 transport function *in vitro* have also been heterogeneous. The key limitations of studies that aim to determine a potential clinical role for OATP2B1 in drug disposition have been the lack of transporter-selective OATP2B1 substrates or inhibitors for use as pharmacological tools. Furthermore, it is possible that the *in vivo* pharmacokinetic effects of functional OATP2B1 genetic variations have been masked or complicated by the fact that altered transport activities in the gut that change oral drug bioavailability may be offset by impacts in other tissues that alter biodistribution and clearance.

In this report we aimed to provide additional insights into the functional consequences of relatively common genetic variants in OATP2B1/*SLCO2B1* by examining potential impacts to endogenous substrate disposition both *in vitro* and *in vivo*. We have shown that the common OATP2B1 c.1457C>T variant has reduced transport activity towards a range of endogenous compounds and a prototypical drug. Importantly, we found associations with the *SLCO2B1* c.935G>A variant with higher plasma concentrations of the endogenous substrates, CPI and CPIII, as well as with greater circulating pregnenolone sulfate levels in individuals carrying the *SLCO2B1* c.1457C>T variant.

In transiently transfected cells, the OATP2B1 c.332G>A, c.601G>A, c.1457C>T variants had the most pronounced effects on OATP2B1 substrate transport, with decreased the cellular accumulation of estrone sulfate, DHEAS, CPI, CPIII and rosuvastatin compared to OATP2B1 wildtype (Figure 2). However, there were substrate-dependenteffects, particularly with the OATP2B1 c.601G>A variant. Reduced transport function of OATP2B1 c.332G>A, c.601G>A and c.1457C>T could be explained by their decreased cell surface expression of OATP2B1 (Figure 3). The OATP2B1 c.332G>A and the c.601G>A variants possessed the highest CADD/REVEL/MetaLR scores (Table 1) among the variants examined and are predicted to change amino acids near or within transmembrane spanning domains of OATP2B1 involved in the substrate translocation pore (Figure 1). Therefore, our results for these variants could be somewhat expected. In the context of previous studies, our observations are consistent with some that found reduced activity of the OATP2B1 c.332G>A and/or c.601G>A variants towards several substrates (Ho et al., 2006) but not with another report that observed no functional effects of the c.601G>Avariant (Nies et al., 2013). On the other hand, the OATP2B1 c.1457C>T variant results in a missense change in an amino acid residue in the large 5th extracellular loop and has a relatively low CADD/REVEL/MetaLR scores. However, we found that the OATP2B1 c.1457C>T variant had reduced transport function *in vitro* which was similar to other studies (Nozawa et al., 2002; Nies et al., 2013). But in contrast, two other studies found increased activity of OATP2B1 c.1457C>T (Ho et al., 2006; Yang et al., 2020). Lastly, we found that the most common OATP2B1 variant, namely c.935G>A, had rather benign functional consequences for substrates, except for a very slight reduction in rosuvastatin transport activity. Such a result would be in keeping with its low CADD/REVEL/MetaLR scores. However, our findings for the OATP2B1 c.935G>A variant contrast with others that find a reduction in transport function for some substrates (Yang et al., 2011; Nies et al., 2013; Yang et al., 2020).

There has been significant interest in circulating endogenous substrates of drug transporters and their potential utility as biomarkers of altered transporter activity. For instance, plasma concentrations of CPI, pyridoxic acid and N1-methylnicontinamide can serve to monitor the activities of OATP1B1/1B3, organic anion transporters (OATs) and organic cation transporters (OCTs), Multidrug And Toxin Extrusion (MATEs), respectively (Ito et al., 2012; Lai et al., 2016; Shen et al., 2019). Pharmacological inhibition or reduced function genetic polymorphisms of these drug transporters could result in elevated plasma concentrations of the endogenous biomarkers through a reduction in systemic clearance conferred by decreased transporter activities in the liver and kidney. Similarly for OATP2B1, we propose that higher concentrations of its endogenous substrates in circulation would signify reduced activity of a OATP2B1 genetic variant.

Estrone sulfate, the most abundant circulating estrogen, is taken up by cells from blood and converted to active estradiol for physiological endocrine function. Estrone sulfate is a well-studied substrate of OATP2B1, however it is also a substrate of many transporters including other OATPs, Na^+^-taurocholate co-transporting polypeptide (NTCP), OATs, organic solute transporter alpha-beta(OSTαβ), BCRP, and MRPs. Consequently, *SLCO2B1* genetic variants were not associated with estrone sulfate plasma concentrations in our cohort of healthy volunteers. This is despite that there was reduced estrone sulfate transport activity with the OATP2B1 c.1457C>T variant *in vitro* and that 17 of 93 participants in the study carried this allele (5 homozygote, 12 heterozygote). However, we did confirm greater estrone sulfate concentrations in individuals with the *SLCO1B1* c.521T>C allele as was previously reported (van der Deure et al., 2008). But the multivariate model for plasma estrone sulfate concentrations was not particularly effective in explaining interindividual variability (R^2^ = 0.047) indicating other genetic and biological factors are important (Platia et al., 1984; Feofanova et al., 2020).

DHEAS and pregnenolone sulfate are circulating sex steroid precursors of androgens and progesterone that are synthesized in the adrenal glands. Intact DHEAS and pregnenolone sulfate are neurosteroid hormones that functionally interact with neurotransmitter receptors and ion channels in the central nervous system (Grube et al., 2018). We observed the well-known and strong relationships between sex and age with plasma DHEAS and pregnenolone sulfate concentrations (Orentreich et al., 1984). DHEAS and pregnenolone sulfate are substrates of similar membrane transporters as estrone sulfate. Indeed, DHEAS is a substrate of OATP1B1/1B3, although previous studies in healthy volunteers found that treatment with rifampin, a potent inhibitor of OATP1B1/1B3, did not affect plasma DHEAS levels (Shen et al., 2017; Takehara et al., 2017). Likewise, we did not find that the reduced function *SLCO1B1* c.521C>T allele was associated with DHEAS (or pregnenolone sulfate) concentrations. But DHEAS and pregnenolone sulfate plasma levels were associated with the *SLCO2B1* variant c.1457C>T in univariate analysis (Table 4). After multivariate regression including the factors of age and sex, DHEAS plasma levels were no longer associated with *SLCO2B1* c.1457C>T. This may be due to the lower age for *SLCO2B1* c.1457C>T carriers compared to those with wildtype *SLCO2B1*. However, with adjustment for age and sex, pregnenolone sulfate concentrations were still predicted to be higher in those carrying *SLCO2B1* c.1457C>T alleles (Table 5). Higher plasma pregnenolone sulfate levels would be consistent with the generally reduced transport activity of the OATP2B1 c.1457C>T variant in our *in vitro* studies.

CPI and CPIII are by-products of heme synthesis that are cleared from the body by biliary and renal excretion, with elimination in bile being the predominant pathway. The hepatocyte uptake of both CPI and CPIII are determined by the actions of OATP1B1, OATP1B3 and OATP2B1, while efflux into bile and blood are dependent on MRP2 and MRP3, respectively (Moriondo et al., 2009; Bednarczyk and Boiselle, 2016; Shen et al., 2016; Kunze et al., 2018). It is notable that while CPI is a good substrate of both OATP1B1 and OATP1B3, it is poorly transported by OATP2B1 (Bednarczyk and Boiselle, 2016; Shen et al., 2016). On the other hand, CPIII is capably transported by OATP1B1, OATP1B3 and OATP2B1 (Bednarczyk and Boiselle, 2016). We also find that OATP2B1 more efficiently transports CPIII than CPI (Figure 2). Genetic mutations that cause combined deficiencies in OATP1B1/OATP1B3 (Rotor Syndrome), result in redirection of CPI and CPIII elimination from bile to urine and an increase in CPI/CPIII urinary ratio (Wolkoff et al., 1976; van de Steeg et al., 2012). Unlike CPI, basal CPIII concentrations in the blood do not appear to be associated with the reduced function *SLCO1B1* c.521T>C allele (Yee et al., 2019). Based on this evidence, we speculated that although CPI and CPIII are both OATP2B1 substrates, circulating CPIII would be more sensitive to the impacts of OATP2B1 genetic variation.

In our cohort of healthy participants, we found that both CPI and CPIII plasma concentrations were significantly influenced by sex and race, but not age. Males had greater concentrations of CPI and CPIII than females by 31 and 28%, respectively. The sex dependency on circulating CPI was previously reported in a cohort of Japanese subjects (Mori et al., 2019) and is thought to be related to differences in synthesis rate (Takita et al., 2020). In univariate analyses, East Asians had greater concentrations of CPI and CPIII compared to Caucasians (Figure 4). However, with multivariable regression, race was no longer an independent predictor of circulating CPI and CPIII (Table 5). It is likely that other covariates, particularly the differing allelic frequencies of *SLCO2B1* variants (c.917G>A, c.935G>A and c.1457C>T) between the subgroups of East Asians and Caucasians (Table 3), largely contributed to the observed racial differences in coproporphyrin concentrations.

The key novel findings of our study are that circulating concentrations of both CPI and CPIII are greater in individuals carrying the most common *SLCO2B1* c.935G>A variant (Table 4). This association was maintained in multiple linear regression when adjusting for other covariates including sex, race, and *SLCO1B1* genotype (Table 5). These results suggest that the *SLCO2B1* c.935G>A variant is a reduced transport function allele *in vivo*. However, this notion is in contrast with the lack of significant functional effects of the OATP2B1 c.935G>A variant observed *in vitro* (Figure 2). We also found that the *SLCO2B1* c.917G>A allele was associated with lower CPIII concentrations (Tables 4, 5). Again, this *in vivo* association was not consistent with our observations of no change in OATP2B1 c.917G>A transport activity *in vitro* (Figure 2). However, it must be cautioned that there were relatively few participants (5 out of 93) with the *SLCO2B1* c.917G>A variant. Another unexpected finding was that the *SLCO2B1* c.935G>A variant was associated with higher plasma CPI concentrations given that CPI is a relatively poor substrate of OATP2B1 and that the absolute hepatic expression of OATP2B1 is approximately one-third of the more efficient CPI transporter, OATP1B1 (Badee et al., 2015). Additionally, we found CPI plasma concentrations were similar between *SLCO1B1* wildtype and *SLCO1B1* c.521T>C variant carriers (TC and CC genotypes), despite other studies having reported increased CPI with the variant allele (Mori et al., 2019; Yee et al., 2019; Suzuki et al., 2021). This difference is likely due to the fact that only one study participant had the homozygous *SLCO1B1* c.521CC genotype, which was previously noted to have the most prominent impacts on CPI levels (Yee et al., 2019; Suzuki et al., 2021). Taken together, our findings imply that both plasma CPI and CPIII are sensitive to alterations in OATP2B1 activity that would be manifest with the possession of functional genetic polymorphisms and during inhibitory drug interactions. It follows that variation in circulating CPI and CPIII concentrations may not distinguish alterations in OATP2B1 activity apart from those occurring for OATP1B1. Finally, it is tempting to speculate that assessment of renal clearance of CPIII could better serve as a selective measure of (renal) OATP2B1 activity since CPIII is highly secreted by the kidney (Lai et al., 2016; Feng et al., 2021), in contrast to CPI which is eliminated mostly by glomerular filtration, and OATP2B1 is expressed in the proximal tubules (Ferreira et al., 2018).

We focused on relatively common missense variants in OATP2B1 to evaluate potential impacts on transporter function both *in vitro* and *in vivo*. However, a recent analysis indicates that rare variation in the *SLCO2B1* gene may account for 11.6% of functional variability in OATP2B1 (Zhang and Lauschke, 2019). Therefore, targeted *in vitro* biochemical evaluation of rare OATP2B1 variants and high-throughput, deep mutational scanning techniques (Zhang et al., 2021), together with case- and population-based association studies are necessary to provide a more complete understanding of the relevance of OATP2B1 genetic variation.

In conclusion, we found that basal circulating concentrations of several endogenous substrates of OATP2B1 were associated with common non-synonymous genetic variations in the transporter in healthy individuals. These genetic associations were poorly aligned with the observed functional activities of the OATP2B1 variants *in vitro*, as well as with predictions from *in silico* algorithms. Additional studies are required to establish whether endogenous substrates may serve as biomarkers of OATP2B1 activity.

## Data Availability

The original contributions presented in the study are included in the article/Supplementary Material, further inquiries can be directed to the corresponding author.
