# Supplementary material for: Organic Anion Transporting Polypeptide 2B1 (OATP2B1) Genetic Variants: In Vitro Functional Characterization and Association With Circulating Concentrations of Endogenous Substrates
Source: Front Pharmacol. 2021 Sep 14;12:713567. doi: 10.3389/fphar.2021.713567 (PMC8476882; doi:10.3389/fphar.2021.713567)

Supplemental TABLE 1 | OATP2B1 variant mutagenesis primers

Gene	Primer Sequence
c.76_84del	5'-TTTGCCTCCAGGTGTGCCCATTGTGGCTTTGG-3'
	5'-CCAAAGCCACAATGGGCACACCTGGAGGCAAA-3'
c.332G>A	5'-GGGGTCGGTGCACCTGGCTGCCAAAATAG-3'
	5'-CTATTTTGGCAGCCAGGTGCACCGACCCC-3'
c.601G>A	5'-CACCCCGCCCATGCCCAGCAGGG-3'
	5'-CCCTGCTGGGCATGGGCGGGGTG-3'
c.917G>A	5'-CCCAAGGAAATGCCCAAGGAAAAACATGAGCTTCAGTTTCGGCG-3'
	5'-GGGTTCCTTTACGGGTTCCTTTTTGTACTCGAAGTCAAAGCCGC-3'
c.935G>A	5'-TGACTGCTAAGACCTTTTGCCGAAACTGAAGCTCA-3'
	5'-TGAGCTTCAGTTTCGGCAAAAGGTCTTAGCAGTCA-3'
c.1457C>T	5'-GCAGCTTGGAAACAGCTCCAGCCCAGGG-3'
	5'-CCCTGGGCTGGAGCTGTTTCCAAGCTGC-3'

Supplemental TABLE 2 | Univariate analyses of other gene variants with circulating endogenous OATP2B1 substrate concentrations.

	Carrier Status	n	Estrone Sulfate (ng/mL)	DHEAS (ng/mL)	Preg. Sulfate (ng/mL)	CPI (nM)	CPIII (nM)
***SLCO1B1* c.388A>G**	NC	30	0.684 (±0.137)	1610 (±240.5)	39.57 (±4.05)	0.774 (±0.060)	0.113 (±0.008)
	C[1]	63	0.751 (±0.104)	1929 (±130.5)	**58.08**** (±3.64)	0.989 (±0.072)	0.121 (±0.006)
***SLCO1B1* c.521T>C**	NC	62	0.667 (±0.106)	1787 (±139.7)	49.11 (±3.43)	0.853 ±0.053)	0.120 (±0.006)
	C	31	0.854 (±0.129)	1904 (±219.5)	58.10 (±5.35)	1.053 (±0.119)	0.115 (±0.008)
***ABCG2* c.421C>A**	NC	68	0.727 (±0.089)	1691 (±134.4)	50.72 (±3.35)	0.870 (±0.063)	0.113 (±0.005)
	C	25	0.736 (±0.195)	2195 (±231.0)	55.88 (±5.94)	1.055 (±0.098)	0.131 (±0.009)
***CYP2C9**2**	NC	77	0.728 (±0.092)	1858 (±135.2)	52.80 (±3.12)	0.886 (±0.048)	0.119 (±0.005)
	C	16	0.734 (±0.192)	1673 (±219.4)	48.80 (±8.06)	1.076 (±0.213)	0.113 (±0.009)
***CYP2C9**3**	NC	88	0.727 (±0.084)	1848 (±122)	52.58 (±3.04)	0.922 (±0.056)	0.120 (±0.005)
	C	5	0.770 (±0.484)	1445 (±458.8)	43.75 (±9.80)	0.885 (±0.171)	0.101 (±0.015)

**ABCC2 c.1249G>A**	NC	62	0.688 (±0.101)	1834 (±149.9)	53.91 (±3.47)	0.902 (±0.052)	0.119 (±0.005)
	C	31	0.813 (±0.145)	1811 (±191)	48.51 (±5.36)	0.955 (±0.124)	0.116 (±0.008)
***ABCC2* c.-24C>T**	NC	56	0.724 (±0.110)	1937 (±153.8)	54.30 (±4.01)	0.975 (±0.079)	0.123 (±0.006)
	C	37	0.743 (±0.128)	1658 (±182.5)	48.79 (±4.13)	0.837 (±0.062)	0.111 (±0.006)

[1]C, Carrier (heterozygotes + homozygotes); NC, non-carriers
Mean ± S.E.M

Supplemental FIGURE 1 | Western Blots Images of OATP2B1 Genetic Variants

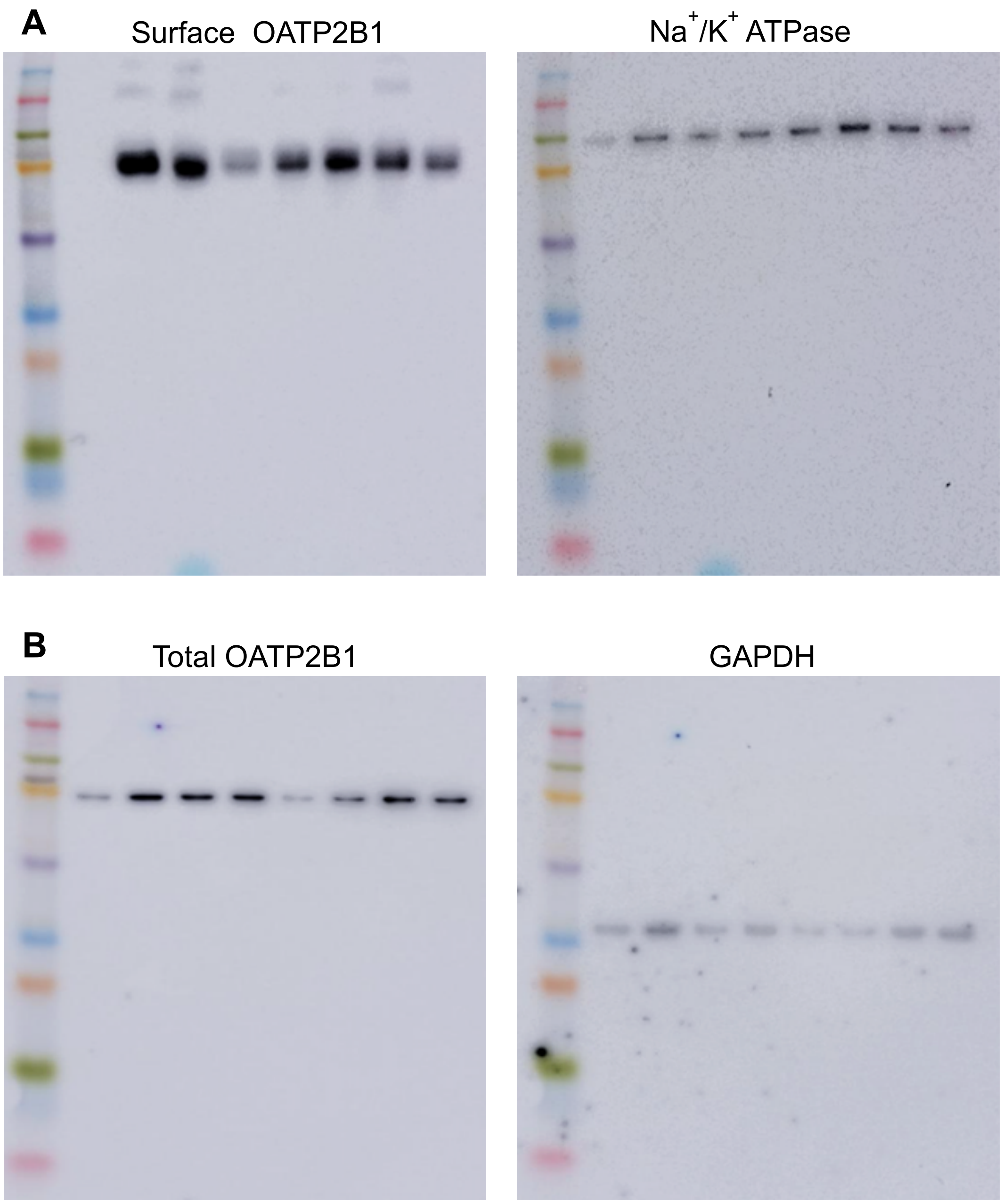

Supplement: Supplementary file 1 [file DataSheet1.PDF]
